# Improved Resolution of 4-Chloromandelic Acid and the Effect of Chlorine Interactions Using (*R*)-(+)-Benzyl-1-Phenylethylamine as a Resolving Agent

**DOI:** 10.3390/molecules23123354

**Published:** 2018-12-18

**Authors:** Yangfeng Peng, Cai Feng, Sohrab Rohani, Quan He

**Affiliations:** 1 School of Chemical Engineering, East China University of Science and Technology, Shanghai 200237, China; cai_erf@126.com; 2 Department of Chemical and Biochemical Engineering, Western University, London, ON N6A 5B9, Canada; srohani@uwo.ca; 3 Department of Engineering, Faculty of Agriculture, Dalhousie University, Truro, NS B2N 5E3, Canada; quan.he@dal.ca

**Keywords:** chlorine interactions, 4-chloromandelic acid, benzyl-1-phenylethylamine, chiral discrimination, resolution

## Abstract

In order to avoid the disadvantage of commonly used resolving agent 1-phenylethylamine (hereafter: PEA), which is soluble in water, (*R*)-(+)-benzyl-1-phenylethylamine ((*R*)-(+)-BPA) was used to resolve 4-chloromandelic acid (4-ClMA) in this study. The optimal resolution conditions were determined: absolute ethanol as a solvent, the molar ratio of 4-ClMA to (*R*)-(+)-BPA as 1:1, the filtration temperature as 15 °C, and the amount of solvent as 1.6 mL/1 mmol 4-ClMA. Thermophysical properties, such as melting point, heat of fusion, and solubility, exhibited significant differences between the less and more soluble salts. The single crystals for the pair of diastereomeric salts were cultivated and their crystal structures were examined thoroughly. In addition to commonly observed interactions like hydrogen bonding and CH/π interactions. The chlorine…chlorine interaction was observed in the less soluble salt presenting as Cl…Cl between adjacent hydrogen network columns, while the Cl/π interaction was observed in the more soluble salt. It was found that halogen interactions played an important role in chiral recognition of 4-ClMA by (*R*)-(+)-BPA.

## 1. Introduction

In response to the new guidelines on drugs with chiral centers launched by the U.S. Food and Drug Administration (FDA) in 1992 [1], significant efforts have been made to develop new methods and/or improve separation efficiency for obtaining enantiopure pharmaceutical intermediates. These methods include catalytic asymmetric synthesis, chromatographic resolution, extraction resolution, membrane resolution, diastereomeric salt resolution, and enzymatic resolution [2,3,4,5,6,7,8] (pp. 9–50). Among these established technologies, optical resolution of racemic substrates through diastereomeric salt formation is still one of the most practical and economical approaches for an industrial scale production, and it has been widely used in the pharmaceutical industry. This route holds a number of advantages, such as the simplicity of crystallization, low cost of operation, recyclability of resolving agents, and availability of both enantiomers. Although extensive experience has been garnered with more than 10,000 successful reported resolutions, the underlying chiral recognition mechanism in the resolution is not well understood. There is no concrete principle to guide the selection and design of resolving agents. For a given racemate, screening the most suitable resolving agent involves tedious trial and error procedures, which is time-consuming and laboratory-intensive [7,8] (pp. 9–50).

Investigation was conducted on the crystal structures of a pair of diastereomeric salts formed between the racemic substrate. The resolving agent is a promising way to study the difference in stability between two salts and gain insights into the chiral discrimination mechanism. The rationale has hinged on an expectation of extracting the common and characteristic crystal structure characteristics of diastereomeric salts in successful resolutions, thus guiding resolving agent selection for a given racemate. Most research efforts have focused on experimentally obtaining crystal structures of diastereomeric salts, followed by qualitative analysis of the difference in molecule packing and intermolecular interactions in the crystal lattices. A number of characteristic features were of great interest, including the structural similarity [9], relative molecular length [10,11,12], hydrogen bonding network [13,14], the CH/π interaction and packing mode of aromatic groups [15,16], and the role of incorporated solvent molecules in diastereomeric salts [17,18]. Recently, two case studies reported the role of halogen bonding interactions in diastereomeric resolution [19,20].

Enantiopure mandelic acid and its derivatives are important chiral building blocks. For example, pure enantiomers of 2-chloromandelic acid (2-ClMA) is used for the production of clopidogrel, a widely administered anticoagulant [21,22]. Enantiopure 4-chloromandelic acid (4-ClMA) is a precursor in the preparation of 2-aryloxy-2-arylalkanoic acids for diabetes and lipid disorders drugs [23]. Phenylethylamine (PEA) is well-recognized as an efficient resolving agent for racemic acidic substrates [24,25]. In ongoing research on the optical resolution of mandelic acid derivatives [26,27,28,29], we had interesting observations: Using PEA as the most commonly used base resolving agent, 4-ClMA was resolved efficiently [26] and 3-ClMA was resolved with a moderate efficiency. However, we failed to resolve 2-ClMA. This indicates that the different position of chlorine in substituted benzene ring led to a significant difference in chiral discrimination. Although PEA is widely used, its solubility in water is relatively high (4% at 20 °C) in the context of optical resolution [30]. This results in a low recovery of PEA in downstream processing. Therefore, an additional extraction step is required [31]. To overcome this challenge, PEA can be modified through the introduction of a benzyl group to its amino group, creating a new resolving agent, *N*-benzyl-1-phenylethylamine (BPA). BPA is proven to be an excellent resolving agent with much lower solubility in water compared to PEA [28]. Since the solubility of BPA in water at 20 °C is only 0.014% [32], BPA can be readily separated from water once the resolution is completed. A high recovery rate of BPA improves the overall economic variability of optical resolution. This encouraged us to further explore a resolution of 4-ClMA using optically active (*R*)-(+)-BPA via diastereomeric salts formation.

To understand the mechanism of chiral discrimination in this new attempt (resolution of 4-ClMA by optical active BPA), the single crystals of both less and more soluble diastereomeric salt, (*R*)-(−)-4-ClMA·(*R*)-(+)-BPA and (*S*)-(+)-4-ClMA·(*R*)-(+)-BPA, were cultivated. From crystal structural examination, it was found that hydrogen bonds, CH/π interaction, van der Waals interaction, supramolecular packing mode, and unique interactions related to the chlorine atom were present in both less soluble and more soluble salts. However, these interactions presented differently. Such interactions of chlorine were not found in the diastereomeric salts from either the resolution of 4-ClMA with PEA [26] or the resolution of 2-chloremandelic acid with BPA [28].

**Figure 1:**
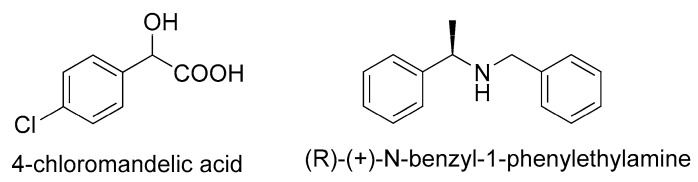
Chemical structure of 4-chloromandelic acid (4-ClMA) and (*R*)-(+)-*N*-benzyl-1-phenylethylamine (BPA).

The chemical structures of 4-ClMA and the resolving agent (*R*)-(+)-BPA are presented in Figure 1.

## 2. Results and Discussion

### 2.1. Solvent Screening and Resolution Condition Determination

As shown in Table 1, racemic 4-ClMA can be resolved by (*R*)-(+)-BPA in most solvents described in 3.7. It is difficult to evaluate the efficiency of a resolution process, as the diastereomeric excess comprises with the yield. The product of diastereomeric excess and yield, termed as optical efficiency (*E*), is sometimes adopted to determine the suitable resolution conditions. The resolution efficiency using 50% ethanol, 2-proponal, acetonitrile, and ethyl acetate is in the range of 87.5–93.2%. This is considered somewhat high. However, considering the low %*d.e*. of diastereomeric salts (52.1% to 55.2%), multiple recrystallizations must be performed to obtain enantiomers with 99 %*e.e*. Therefore, ethanol with a relatively high %*d.e.* is chosen as a solvent for the following investigation. Additional benefit of using ethanol is its low price compared to other solvents.

Once a suitable solvent was identified, other factors affecting the resolution process were investigated, including the mole ratio of 4-ClMA to (*R*)-(+)-BPA, the amount of ethanol solvent, and the filtration temperature. In order to reduce experiment runs, a three-factor and three-level orthogonal experiment design, 3^3^, was used. Table S1 summarized the experimental results. Based on the orthogonal design analysis, the optimal resolution conditions were determined to be the molar ratio of 4-ClMA to (*R*)-(+)-BPA of 1:1, the amount of absolute ethanol of 1.6 mL/1 mmol 4-ClMA, and the filtration temperature of 15 °C. The optimal conditions derived from orthogonal design were verified by experiments. The resolution efficiency *E* reached 84.3% under such conditions, which is significantly higher than the resolution efficiency *E* of 71.4% in the resolution of (*R*,*S*)-4-ClMA by (*R*)-(+)-PEA, as reported in the literature [26].

### 2.2. Thermodynamic Properties of Diastereomeric Salts

To explore the chiral recognition ability of (*R*)-(+)-BPA, the corresponding less and more soluble salts, (*R*)-(−)-4-ClMA·(*R*)-(+)-BPA and (*R*)-(−)-4-ClMA·(*S*)-(−)-BPA, were synthesized as described in Section 3.3 and Section 3.4. It is generally common that an efficient separation can be expected when the difference in melting point of a pair of diastereomeric salt is larger than 20 °C. The trends of solubility difference between diastereomeric salts parallels include differences in melting points and heat of fusion [33,34]. The thermal properties of the resulting salts were determined and listed in Table 2. The melting point and solubility differences were significant. The melting point of the less soluble salt exceeded that of the corresponding more soluble salt by 34.3 °C. The difference in heat of fusion between the less and more soluble salt was 4.83 kJ·mol^−1^. Table 3 also showed that the solubility ratio of the more soluble salt (*R*)-(−)-4-ClMA·(*S*)-(−)-BPA to less soluble salt (*R*)-(−)-4-ClMA·(*R*)-(+)-BPA in absolute ethanol at 20 °C is 3.3:1.

The comparison of the aforementioned thermal properties strongly suggests that the less soluble salt is significantly more stable than the more soluble salt, and the high-resolution efficiency achieved in the Section 2.1 resulted from such difference in stability.

**Figure 2:**
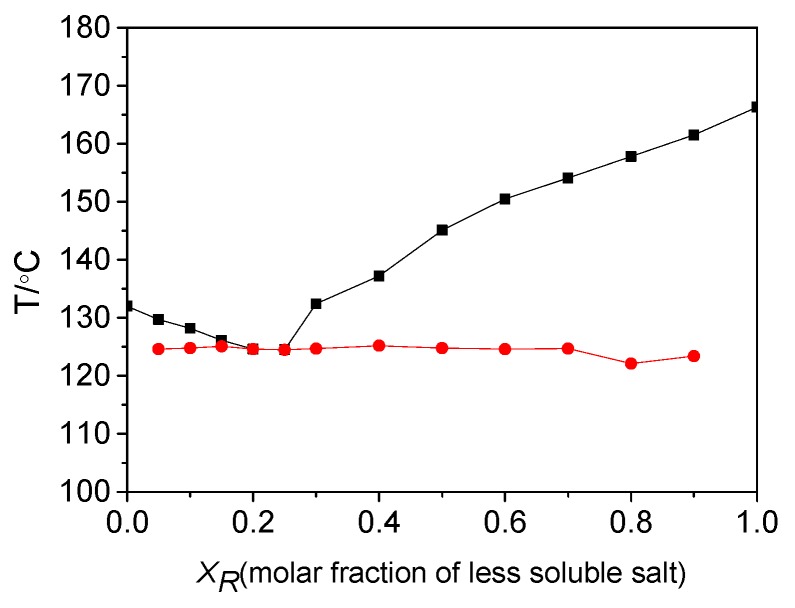
Binary melting point phase diagram of diastereomeric salts. The circle and square represent the temperatures at the beginning and the end of fusion, respectively.

A binary melting point phase diagram of diastereomeric salts was also established. In general, diastereomeric salt mixtures are classified into three types: A eutectic conglomerate, a 1:1 addition compound, and a solid solution [33,34,35,36]. The type of diastereomeric salt mixture can be identified on the basis of a binary melting point phase diagram. The formation of eutectic conglomerate is the primary condition for resolution. Figure 2 shows the binary melting point phase diagram for a mixture of (*R*)-(−)-4-ClMA·(*R*)-(+)-BPA and (*R*)-(−)-4-ClMA·(*S*)-(−)-BPA. This mixture was constructed by a detailed DSC analysis of mixtures with different compositions of *X_R_*, which was the fraction of more soluble salt (*R*)-(−)-4-ClMA·(*R*)-(+)-BPA. The system was a eutectic mixture, and the eutectic composition, *X_e_*, was 0.25 from experimental results, which is in agreement with the *X_e_* of 0.25, calculated theoretically from thermodynamic Schroder-van-Laar equation [37]. The fact that the system is a eutectic mixture indicates that the resolution efficiency primarily depends on the difference in stability between the less and more soluble salts.

To further the understanding of the stability difference between the pair of diastereomeric salts of BPA and 4-ClMA, the single crystals of the corresponding diastereomeric salts were grown and examined by X-ray diffraction.

### 2.3. Crystal Structure of Diastereomeric Salts

**Figure 3:**
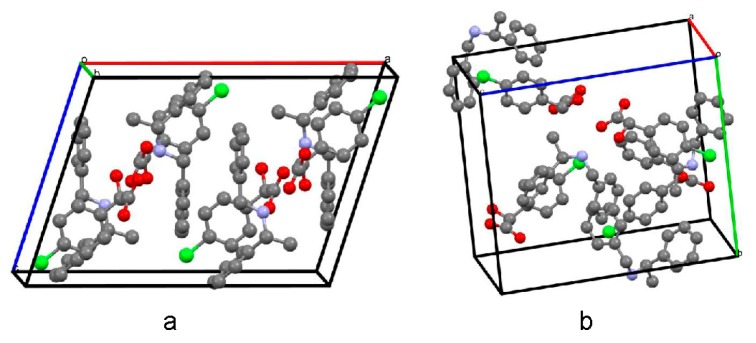
Molecules packing in the unit cells of (*R*)-(−)-4-ClMA·(*R*)-(+)-BPA (**a**) and (*S*)-(+)-4-ClMA·(*R*)-(+)-BPA (**b**).

High-quality single crystals of the less soluble salt (*R*)-(−)-4-ClMA·(*R*)-(+)-BPA were readily produced in the solvent of 2-propanol. The single crystal of the more soluble salt (*S*)-(+)-4-ClMA·(*R*)-(+)-BPA was obtained in a co-solvent solution of acetonitrile and methanol. Crystals of (*R*)-(−)-4-ClMA·(*R*)-(+)-BPA were colorless rods crystallized in a monoclinic C2 space group (Appendix A, ccdc 1030316). Each unit cell contained four (*R*)-(−)-4-ClMA anions and (*R*)-(+)-BPA cations. Crystals of (*S*)-(+)-4-ClMA·(*R*)-(+)-BPA were colorless plates crystallized in an orthorhombic P2_1_2_1_2_1_ space group (Appendix A, ccdc 1885316). Each unit cell contained four (*S*)-(+)-4-ClMA anions and (*R*)-(+)-BPA cations, as shown in Figure 3. Figure S1 graphically displayed the atomic numbering schemes. Detailed crystal data of (*R*)-(−)-4-ClMA·(*R*)-(+)-BPA and (*S*)-(+)-4-ClMA·(*R*)-(+)-BPA were summarized in Table S2.

The arrangement and stacking modes of the organic molecules in the crystal structure mainly depends on a number of weak intra and/or intermolecular interactions [38,39,40,41]. In the context of an optical resolution through diastereomeric salt formation, a basic compound and an acidic compound can form various kinds of crystals depending on their molecular structures and functional groups, respectively. In the process of crystallization, it is well-recognized that these molecules arrange as compactly as possible through interactions like hydrogen bonding, CH/π, and van der Waals force to realize the minimization of energy [9,10,11,12,13,14,15,16,17,18]. The hydrogen bond was conventionally considered to be the most important factor in accounting for chiral discrimination because it is stronger than other interactions in determining the crystal structure. CH/π interaction, which involves a weak hydrogen bond, has been considered a secondary interaction in comparison with hydrogen bonds. However, accumulated evidence indicates that CH/π interaction in diastereomeric salt crystal structures may contribute to chiral discrimination to a considerable extent. Recently, other intermolecular interactions, like halogen bonds, were found to contribute to chiral discrimination [19,20]. The difference in the stabilities of diastereomeric salts come from such super-molecular interactions. Therefore, they were examined in detail as follows.

#### 2.3.1. Hydrogen-Bonding Network

Table 3 and Table 4 show the hydrogen-bonding geometry of (*R*)-(−)-4-ClMA·(*R*)-(+)-BPA and (*S*)-(+)-4-ClMA·(*R*)-(+)-BPA, respectively.

In the single crystal of the less soluble salt (*R*)-(−)-4-ClMA·(*R*)-(+)-BPA, two intramolecular hydrogen bonds were found: N1B-H1BB…O1A and N1B-H1BB…O3A. There also were three intermolecular hydrogen bonds: N1B-H1BA…O2A1, O3A-H3A…O2A1, and O3A-H3A…O1A1. The two intramolecular hydrogen bonds were fundamental for the formation of less soluble salts. Three intermolecular hydrogen bonds, one from the ammonium hydrogen of (*R*)-(+)-BPA and neighboring carboxylic oxygen of (*R*)-(−)-4-ClMA and the other two from the carboxylic hydrogens of (*R*)-(−)-4-ClMA and the adjacent carboxylic oxygens, formed the hydrogen network along the C-axis. As shown in Figure S2, such intermolecular hydrogen bonds formed the one-dimensional (1D) double-helix chains in the H-bonding network. These results are similar to those reported in the resolution of 2-ClMA with BPA [28], and different from those formed between primary amines and carboxylic acids [13,26,27].

There are three types of hydrogen bonds present in crystal structure of the more soluble salt of (*S*)-(+)-4-ClMA·(*R*)-(+)-BPA. Two were ammonium hydrogens of (*R*)-(+)-BPA with carboxylate oxygens from adjacent (*S*)-(+)-4-ClMA molecules: N1-H1A…O1 and N1-H1B…O2. One was O3-H3…O1, which was from the hydroxygen hydrogen of (*S*)-(+)-4-ClMA and carboxylate oxygen from the neighboring (*S*)-(+)-4-ClMA. The H-bonding network also presented a pattern of 1D double-helix chains. There was no significant difference observed in the H-bonding networks between the less and more soluble salts, implying that chiral recognition did not primarily originate from hydrogen bond motifs.

#### 2.3.2. CH/π and π/π Interaction

CH/π interaction is considered a weak hydrogen bond and plays an important role in areas such as crystal packing and molecular recognition. The contribution of the CH/π interaction to chiral discrimination was recently recognized [15,16,27,42,43]. In this study, we found CH/π interactions not only within the hydrogen bonding columns, but in adjacent hydrophobic layers in the crystal structure of the less soluble salt (*R*)-(−)-4-ClMA·(*R*)-(+)-BPA. Specifically, as shown in Figure S3, CH/π interactions were present between the benzene ring of C3-C4…C8 of (*R*)-(−)-4-ClMA and the CH groups C2B-H2B and C5B-H5B at neighboring benzene rings of (*R*)-(+)-BPA within the hydrogen-bonding network columns. The CH/π distances were 2.624 Å and 2.740 Å, respectively. In Figure S4, the CH group at benzyl group of (*R*)-(+)-BPA in one hydrophobic layer interacted with the benzene ring of benzyl group of (*R*)-(+)-BPA from an adjacent layer. The CH/π distance was 2.847 Å. Additionally, π/π interactions were observed. Interactions formed between the two benzene rings of (*R*)-(+)-BPA from the two hydrophobic layers with a distance of 3.365 Å.

However, in the crystal structure of the more soluble salt (*S*)-(+)-4-ClMA·(*R*)-(+)-BPA, no such CH/π interactions were discovered, indicating that the CH/π interactions played a dominant role in realizing the close packing among hydrophobic layers. This conclusion is consistent with the observations reported in other studies [15,16,27,28].

#### 2.3.3. Chlorine…Chlorine/π Interactions

Excluding hydrogen bonds and CH/π interactions, there are other weak interactions in crystal structure of salts, such as halogen bonds. Halogen-bonding is a type of weak interaction, and its strength is comparable to that of hydrogen bonding. The halogen bond has an important role in molecular assembly in many areas, such as crystal engineering, material science, molecular recognition, medical design, and organic reaction [40,41]. Although halogen-bonding has attracted much attention in supramolecular chemistry, there is limited research regarding the role of halogen bonds in the chiral discrimination of optical resolutions. In 1999, Farina resolved 1,2-dibromohexfluoropropane with sparteine in the solvent of chloroform, and halogen bonds were first observed to contribute to the success of this resolution [20]. Kobayashi et al. purposely designed racemic substrates and resolving agents with halogen atoms and found that halogen-bonding interactions could effectively stabilize diastereomeric salt crystals [19]. In addition, there are Cl-Cl or Cl/π interactions that cannot be considered halogen bonds [40,41,44]. However, they play similar roles in the assembly of molecules. In this study, chlorine interactions similar to halogen bonds were observed. They presented differently in less soluble and more soluble salts. This chlorine-involved interaction in the less soluble salt was the Cl…Cl type with a distance of 3.193 Å, as shown in Figure S5. This distance was smaller than the addition of van der Waal radii of two chlorine atoms (2 × 1.75 Å). Both angles of the C-Cl1…Cl2 and the Cl1…Cl2-C were 165.27°, forming a type I Cl…Cl interaction, but only type II are defined as halogen bonds [40]. This Cl…Cl interaction connected the hydrogen-bonding network columns A and C, or B and D, which helped realize a compact packing of these columns. This was due to the chlorine of the A or B columns, located exactly opposite the neighboring chlorine of the C or D columns. Therefore, a type I Cl…Cl interaction was found to contribute to chiral recognition.

In the more soluble salt (*S*)-(+)-4-ClMA·(*R*)-(+)-BPA, there was another weak interaction, Cl/π, with a distance of 3.328 Å, as shown in Figure S6. This interaction was formed between the chlorine of (*S*)-(+)-4-ClMA and the benzene group of (*R*)-(+)-BPA from the adjacent column, and connected the hydrogen-bonding network columns A and B, or C and D. C-X…π halogen bonds have been reported in the literature, in which halogen was connected to aliphatic hydrocarbon [45,46], forming C-X/π angle of 180 °C. However, in the more soluble salt, Cl was connected to the benzene ring, as shown in Figure S6. The angle of C21-Cl1…C13 was 159.52°, while the angle of C-Cl…π was smaller than 159.52°, both of which were far from 180°.

Generally, the length (3.193 Å) of the Cl…Cl interaction in the less soluble salt was shorter than that (3.328 Å) in the more soluble salt, and significantly shorter than those of halogen bonds (3.3 Å to 3.46 Å) reported in the literature [19,20]. This favored a compact packing of the less soluble salt in addition to the contribution from CH/π or π/π interactions, discussed in Section 2.3.2. The combined effects of halogen interactions, CH/π and/or π/π interactions significantly increased the stability of the less soluble salts, and eventually facilitated an efficient chiral discrimination in the resolution of 4-ClMA with (*R*)-(+)-BPA.

#### 2.3.4. Stacking Mode

In the hydrophobic region, the packing of aromatic groups of less soluble salt (*R*)-(−)-4-ClMA·(*R*)-(+)-BPA and more soluble salt (*S*)-(+)-4-ClMA·(*R*)-(+)-BPA was different, as shown in Figures S7 and S8, respectively.

In the less soluble salt, the benzene rings of (*R*)-(−)-4-ClMA were arranged in T-shapes relative to the neighboring benzene ring of (*R*)-(+)-BPA with an interplanar angle of 93.63°. This type of T-shaped packing of aromatic groups has been well-recognized as energetically favorable to crystal stability [39]. It resulted in a closer packing of columns and benefited the CH/π hydrogen-bonding interaction between columns in the less soluble salts. The benzene rings from (*R*)-(−)-4-ClMA were parallel to one another with a distance of 9.699 Å. This distance is not short enough to account for π/π interactions. Neither were the benzene rings of (*R*)-(+)-BPA. Between hydrophobic layers, a “key and lock” pattern was observed, as shown in Figure S7b. Generally, a planar boundary surface between hydrophobic layers is favorable, since it is able to tightly stack these layers and stabilize the crystal structure [9,11,13]. Unfortunately, in this study, the molecular length of BPA was significantly longer than that of 4-ClMA. Thus, a corrugated boundary surface was formed. The molecules were arranged in a “key and lock” mode, minimizing steric hindrance. This observation is similar to the packing mode in the resolution of 2-ClMA and (*R*)-(+)-BPA [28].

In the more soluble salts, no T-shaped arrangements of aromatic groups were found. The benzene rings of (*R*)-(−)-4-ClMA inclined to the neighboring benzene ring of (*R*)-(+)-BPA. The benzene rings from (*R*)-(−)-4-ClMA were parallel to one another with a distance of 9.179 Å, as shown in Figure S8. A “key and lock” mode was also formed, and the boundary surface was corrugated. Clearly, the “key and lock” packing in the more soluble salt was less compact compared to that in the less soluble salt. A relatively larger space and more voids were seen.

## 3. Materials and Methods

### 3.1. Materials

(*RS*)-4-ClMA with a purity of 99% was purchased from Alfa Aesar Johnson Matthey Company (WardHill, MA, USA). ®-(+)-BPA and (*S*)-(−)-BPA, with an optical purity of 99%, were purchased from Aldrich-Sigma Canada (Oakville, ON, Canada). All solvents were analytically pure, as purchased from Aldrich-Sigma Canada or Tiechem (Shanghai, China). The methanol/acetonitrile used for HPLC was HPLC grade and was purchased from Tiechem. Hydroxypropyl-β-cyclopram was purchased from Shandong Binzhou Zhiyuan Biotechnology Company Ltd. (Binzhou, China).

### 3.2. Analytical Methods

The melting points and heat of fusion of diastereomeric salts were determined by Mettler Toledo DSC 822 (Grei-fensee, Switzeland). The samples (3–6 mg) were prepared in a covered aluminum crucible with a pierced lid to allow the escape of volatiles. The sensors and samples were under nitrogen purge during the experiments. A heating rate of 5 °C/min was employed. The optical purity for the less soluble salt of (*R*)-(−)-4-ClMA·(*R*)-(+)-BPA and more soluble salt of (*R*)-(−)-4-ClMA·(*S*)-(+)-BPA or (*S*)-(−)-4-ClMA·(*R*)-(+)-BPA were determined t 25 °C by a reverse-phase HPLC equipped with a cyanopropyl column (UF-CN: 220 × 4.6 mm × 5 μm, Zhongpu Sci. Dalian, China), with hydroxypropyl-β-cyclodextrin as a chiral mobile phase additive. The HPLC system (Hegong Instrument, Shanghai, China) consisted of a Vertex STI 5000 pump, a STI UV detector (wavelength range of 190–700 nm), a 10 µL sample loop (Rheodyne), and a 7725i sampler. The mobile phase was a mixture of an aqueous buffer (pH of 2.8, 8 mmol/L sodium dihydrogen phosphate and 5 mmol/L hydroxypropyl-β-cyclodextrin) and methanol with a volumetric ratio of 95:5. The flow rate was 1.0 mL/min and the detector wavelength was set at 220 nm.

Crystal structures of the diastereomeric salts were determined by Single Crystal X-ray Diffraction. X-ray crystallography for the less soluble salt (*R*)-(−)-4-ClMA·(*R*)-(+)-BPA was carried out at the Western University in London, Ontario, Canada. Data collection: APEX2 (Bruker, Karlsruhe, Germany, 2009). Cell refinement: SAINT (Bruker, 2009); Data reduction (SAINT); Programs used to solve structure: SHELXT (Sheldrick, Göttingen, Germany, 2008); Programs used to refine structure: SHELXL2014 (Sheldrick, 2008); Molecular graphics: PLATON (Spek, Utrecht, The Netherlands, 2009). X-ray crystallography for the more soluble salt (*S*)-(−)-4-ClMA·(*R*)-(+)-BPA was carried out at Fudan University in Shanghai, China. Data collection: Bruker SMART; Cell refinement: Bruker SMART; Data reduction: Bruker SHELXTL; Programs used to solve structure: Bruker SHELXTL; Programs used to refine structure: SHELXL-2014/7 (Sheldrick, 2014); Molecular graphics: Bruker SHELXTL.

### 3.3. Preparation of the Less Soluble Salt (R)-(−)-4-ClMA·(R)-(+)-BPA

The less soluble salt was synthesized by enantiopure (*R*)-(−)-4-ClMA and (*R*)-(+)-BPA. (*R*)-(+)-BPA (1.2 mL, 5.9 mmol) was added dropwise to a solution of (*R*)-(−)-4-ClMA (1.1 g, 5.9 mmol) in 20 mL 2-propanol, forming white crystals. The slurry was heated to reflux and was kept at the temperature for 15 min. Subsequently, the mixture was cooled to room temperature. Then, 5 mL hexane was added to the mixture to suspend the solid, and the crystals were collected by filtration and washed with 2-propanol (1.5 mL × 2) twice to give 2.0 g enantiopure (*R*)-(−)-4-ClMA·(*R*)-(+)-BPA with a yield of 85%. The melting point of the salt was 166 °C, specific rotation ${\left[\alpha \right]}_{D}^{22}=-32.0{}^{\circ}$ (c = 1, methanol). ^1^HNMR (400 MHz, DMSO) δ: 1.37–1.39 (d, 3H, CH_3_), 2.50 (s, 2H, CH_2_), 3.91–3.96 (m, 1H, CH), 4.85 (s, 1H, CH), 7.27–7.44 (m, 14H, C_6_H_4_ + C_6_H_5_ + C_6_H_5_). Elemental analysis: Calculated for C_23_H_24_ClNO_3_ (FW397.88) C: 69.43, H: 6.04, N: 3.52; Found C: 69.26, H: 6.24; N: 3.42.

### 3.4. Preparation of the More Soluble Salt (R)-(−)-4-ClMA·(S)-(−)-BPA

The more soluble salt (*R*)-(−)-4-ClMA·(*S*)-(−)-BPA was synthesized by optical purity (*R*)-(−)-4-ClMA and (*S*)-(−)-BPA. (*R*)-(+)-BPA (1.1 g, 5.0 mmol) was added dropwise to a solution of (*R*)-(−)-4-ClMA (0.935 g, 5.0 mmol) in 6 mL 2-propanol, and the white crystals appeared. The slurry was heated to 65 °C, the solid was dissolved completely. The solution was kept at 65 °C for 0.5 h, then cooled slowly to 20 °C. The crystals were collected by filtration and washed with 2-propanol (0.75 mL × 2) twice to give 1.72 g enantiopure (*R*)-(−)-4-ClMA·(*S*)-(−)-BPA with a yield of 86.3%. Its melting point was 138.1 °C; specific rotation ${\left[\alpha \right]}_{D}^{22}=-51.1{}^{\circ}$ (c = 1, methanol), ^1^HNMR (400 MHz, DMSO) δ: 1.36–1.38 (d, 3H, CH_3_), 2.50 (s, 2H, CH_2_), 3.92–3.94 (m, 1H, CH), 4.84 (s, 1H, CH), 7.21–7.43 (m, 14H, C_6_H_4_ + C_6_H_5_ + C_6_H_5_). Elemental analysis: Calculated for C_23_H_24_ClNO_3_ (FW397.88) C: 69.43, H: 6.04, N: 3.52; Found C: 69.39, H: 6.25; N: 3.42. The more soluble salt (*S*)-(+)-4-ClMA·(*R*)-(+)-BPA, the enantiomer of (*R*)-(−)-4-ClMA·(*S*)-(−)-BPA, was prepared in a similar way, its melting point was 131.2 °C, and specific rotation ${\left[\alpha \right]}_{D}^{22}=+51.4{}^{\circ}$ (c = 1, methanol).

### 3.5. Solubility Determination

The solubilities of less and more soluble salts were measured by a synthetic method [47]. The diastereomeric salts were weighed and added in the barrel-type test tube with a thermometer, then the tube was set in a jacket glass flask with a temperature control. A certain amount of absolute ethanol solvent was added dropwise into the tube, and the mixture was stirred at a constant temperature of 20 °C for at least 15 min. Ethanol was continuously added until the diastereomeric salts were completely dissolved. The quantity of added absolute ethanol were weighed accurately to determine the solubilities of two diastereomeric salts. The measurement was performed in duplicate and the average value was reported in this paper. The accuracy of balance was ±1 mg.

### 3.6. Determination of Binary Phase Diagram of Diastereomeric Salts

A weighed amount of pure less soluble salt (*R*)-(−)-4-ClMA·(*R*)-(+)-BPA and more soluble salt (*R*)-(−)-4-ClMA·(*S*)-(−)-BPA were mixed in a mortar and dissolved in a small amount of absolute methanol. The solid appeared in the mortar and the slurry was crushed until the methanol evaporated completely to give a uniform mixture with a different diastereomeric composition. The resolution of the electronic balance was 0.01 mg. The binary melting point phase diagram was established by using Mettler Toledo DSC 822e differential scanning calorimeter (Greifense, Switzerland) by measuring the temperature at the beginning and the end of fusion of diastereomeric salt mixtures.

### 3.7. Resolution Procedure

A typical resolution process is described as follows: 4-ClMA (0.935 g, 0.005 mol) and 8 mL absolute ethanol were mixed in a 20 mL bottle under magnetic stirring at room temperature to form a complete dissolution of 4-ClMA. (*R*)-(+)-BPA (1.05 g, 0.005 mol) was added dropwise into the solution and white crystals, namely diastereomeric salts, soon appeared. The mixture was heated by a water bath to 72 °C and stayed at the temperature for 15 min, and the solid was completely dissolved. The solution was then cooled slowly to 20 °C and stayed for 15 min, the precipitated crystalline salts were collected by filtration and washed with cold absolute ethanol (1 mL × 2) twice. The obtained diastereomeric salt was 0.82 g with a yield of 81.8% (on the basis of (*R*)-(−)-4-ClMA·(*R*)-(+)-BPA in the mixture). Its melting point was 164.6 °C, specific rotation ${\left[\alpha \right]}_{D}^{22}=-27.7{}^{\circ}$ (c = 1, methanol), the optical purity was 94.8 %*d.e.* based on the following formula.

(1) $$\mathrm{Diastereomeric}\;\mathrm{excess}:\;\%d.e.=\frac{{\left[\alpha \right]}_{D}^{22}-51.1{}^{\circ}}{-32.0{}^{\circ}-51.1{}^{\circ}}$$

Here, ${\left[\alpha \right]}_{D}^{22}$ represents the specific rotation of the precipitated salts from a resolution process, the specific rotation of (*R*)-(−)-4-ClMA·(*R*)-(+)-BPA (less soluble salt) was −32.0°, and that of (*R*)-(−)-4-ClMA·(*S*)-(−)-BPA (more soluble salt) was −51.1°. The optical purity calculated by the formula above was in a good agreement with the optical purity determined on a chiral column using HPLC. Therefore, diastereomeric purity of the salts determined by the specific rotation was used in this study due to the convenience in its measurement compared to the measurement using HPLC.

## 4. Conclusions

In the resolution of 4-Chloromandelic acid with (*R*)-(+)-benzyl-1-phenylethylamine, the resulting less soluble and more soluble diastereomeric salts exhibited significant differences in their thermodynamic properties, including melting points of 166.3 °C and 132.0 °C, enthalpy of fusion of 57.41 KJ/mol and 52.58 KJ/mol, and solubility of 1.47 g/100 g ethanol and 4.82 g/100 g ethanol, respectively. These differences originated from the distinct supramolecular interactions in the crystal lattice of the pair of diastereomeric salts. In addition to well-recognized hydrogen-bonding, CH/π interactions and aromatic groups packing, halogen involved interactions, such as Cl…Cl and Cl/π. These interactions were observed, and they demonstrated significant contributions to chiral discrimination.

## Figures and Tables

**Table 1:** Solvent screening on the resolution of (*R*,*S*)-4-ClMA by (*R*)-(+)-BPA.

4-ClMA/mol	BPA/mol	Solvent ^a^	*%d.e.*	Yield ^b^/%	*E*^c^/%
0.005	0.005	Methanol	94.5	55.9	52.8
0.005	0.005	Ethanol	94.8	83.1	78.8
0.005	0.005	95% ethanol	96.3	76.3	73.5
0.005	0.005	50% ethanol	55.2	158.5	87.5
0.005	0.005	2-propanol	56.3	156.9	88.4
0.005	0.005	Acetonitrile	52.1	172.0	89.6
0.005	0.005	Ethyl acetate	52.8	176.4	93.2
0.005	0.005	chloroform	No salts		

^a^ The volume of all solvents used here was 8 mL. ^b^ The yield was equal to and resulted at the temperature of filter of 20 °C. ^c^ Resolution efficiency *E* is the product of yield and diastereomeric purity.

**Table 2:** Thermal properties of diastereomeric salts of (*R*,*S*)-4-ClMA and (*R*)-(+)-BPA.

	Solubility ^a^/g	Melting Point/°C	Heat of fusion/kJ·mol^−1^
Less soluble salt	1.47	166.3	57.41
More soluble salt	4.81	132.0	52.58

^a^ Weight (g) of the solute dissolved in 100 g absolute ethanol solvent at 20 °C.

**Table 3:** Hydrogen-bonding geometry of (*R*)-(−)-4-ClMA·(*R*)-(+)-BPA [Å and °].

D-H…A	d(D-H)	d(H…A)	d(D…A)	<(DHA)
O3A-H3A…O1A ^a^	0.87(2)	1.84(2)	2.6878(15)	164.9(17)
O3A-H3A…O2A ^a^	0.87(2)	2.52 (2)	3.1629(14)	130.6(16)
N1B-H1BA…O2A ^a^	0.85(2)	1.90(2)	2.7457(16)	176.1(16)
N1B-H1BB…O1A	0.98(2)	1.78(2)	2.7337(15)	163.7(18)
N1B-H1BB…O3A	0.98(2)	2.42(2)	3.0019(14)	117.4(15)

Symmetry codes: (a) −x + 3/2, y − 1/2, −z + 1.

**Table 4:** Hydrogen-bonding geometry of (*S*)-(+)-4-ClMA·(*R*)-(+)-BPA [Å and °].

D-H…A	d(D-H)	d(H…A)	d(D…A)	<(DHA)
O(3)-H(3)...O(1) ^a^	0.90(3)	1.81(3)	2.707(3)	170(3)
N(1)-H(1A)...O(1) ^b^	1.04(3)	1.84(3)	2.867(3)	169(2)
N(1)-H(1B)...O(2) ^c^	0.93(3)	1.80(3)	2.690(3)	158(3)

Symmetry codes: (a) x − 1/2, −y + 1/2, −z + 1; (b) x,y + 1,z; (c) x − 1/2, −y + 3/2, −z + 1.

## Supplementary Materials

The following are available online, Table S1: The Orthogonal Experiment Result for the Resolution of (*R*,*S*)-4-ClMA by (*R*)-(+)-BPA; Table S2: Crystal Structure Data of (*R*)-(−)-4-ClMA·*(R)*-(+)-BPA and (*S*)-(+)-4-ClMA·*(R)*-(+)-BPA; Figure S1: Atomic-numbering Schemes of (*R*)-(−)-4-ClMA·*(R)*-(+)-BPA (a) and (*S*)-(+)-4-ClMA·*(R)*-(+)-BPA (b); Figure S2: The H-bonding network in the less soluble salt (a and b) and more soluble salt (c and d). The red parts represent carboxylate anions of 4-ClMA and the blue parts represent ammonium cations of BPA; Figure S3: The CH/π interactions within hydrogen column of less soluble salt; Figure S4: The CH/π interactions between adjacent hydrophobic layers of less soluble salt. Viewed from b-axis.; Figure S5: The Cl…Cl interactions between adjacent hydrogen bonding net work columns and the view of adjacent four columns from b-axis in the less soluble salt (a) viewed from a-axis; (b) viewed from b-axis; Figure S6: The Cl/πhalogen bonds interactions between columns and the view of adjacent four columns from a-axis in the more soluble salt (a) viewed from b-axis; (b) viewed from a-axis.; Figure S7: Packing mode of the less soluble salt in hydrophobic region, (a) The distance of benzene rings, (b) the packing mode of hydrophobic layers (viewed from b-axis); Figure S8: Packing mode of the more soluble salt in hydrophobic region, a. The distance between benzene rings, b. the packing mode of hydrophobic layers (viewed from b-axis).

## Appendix A

The crystal data of (*R*)-(−)-4-ClMA·(*R*)-(+)-BPA and (*S*)-(+)-4-ClMA·(*R*)-(+)-BPA is available for reference at CCDC 1030316 and 1885316.
