# Supplementary material for: Improved Resolution of 4-Chloromandelic Acid and the Effect of Chlorine Interactions Using (R)-(+)-Benzyl-1-Phenylethylamine as a Resolving Agent
Source: Molecules. 2018 Dec 18;23(12):3354. doi: 10.3390/molecules23123354 (PMC6320892; doi:10.3390/molecules23123354)
Supplement: Supplementary file 1 [file molecules-23-03354-s001.pdf]

Supplementary Materials

Improved Resolution of 4-Chloromandelic Acid and the Effect of Chlorine Interactions Using (*R*)-(+)-Benzyl-1-Phenylethylamine as a Resolving Agent

Yangfeng Peng [1,*], Cai Feng [1], Sohrab Rohani [2], and Quan (Sophia) He [3]

[1] School of Chemical Engineering, East China University of Science and Technology, Shanghai 200237, China; yfpeng@ecust.edu.cn
[2] Department of Chemical and Biochemical Engineering, Western University, London, ON N6A 5B9, Canada; srohani@uwo.ca
[3] Department of Engineering, Faculty of Agriculture, Dalhousie University, Truro, NS B2N 5E3, Canada; quan.he@dal.ca
* Correspondence: yfpeng@ecust.edu.cn; Tel: +86-21-64252345

Table S1. The Orthogonal Experiment Result for the Resolution of (*R*, *S*)-4-ClMA by (*R*)-(+)-BPA.

Entry	$n_{4\text{-}ClMA}$:n_{BPA}	*T*/°C	*V*/ml	d.e./%	Yield/%	*E*/%
1	1：1	25	10	95.4	78.1	74.5
2	1：1	20	6	95.6	84.8	81.1
3	1：1	15	8	94.8	88.9	84.3
4	1：0.85	25	6	95.0	81.7	77.6
5	1：0.85	20	8	96.4	80.3	77.4
6	1:0.85	15	10	97.9	79.9	78.2
7	1:0.70	25	8	96.8	70.8	68.5
8	1:0.70	20	10	95.4	71.9	68.6
9	1:0.70	15	6	95.0	79.9	75.9
X_{1j}	71.00	78.20	79.47		X=76.23	
X_{2j}	77.73	76.73	75.70			
X_{3j}	79.97	73.77	73.53			
SS_j	43.59	10.18	18.06		SS_T=71.83	

4-ClMA=0.005mol in all cases.

Table S2. Crystal Structure Data of (*R*)-(-)-4-ClMA·(*R*)-(+)-BPA and (*S*)-(+)-4-ClMA·(*R*)-(+)-BPA.

	**(*R*)-(-)-4-ClMA·(*R*)-(+)-BPA**	**(*S*)-(+)-4-ClMA·(*R*)-(+)-BPA**
Empirical formula	$C_{23}H_{24}ClNO_3$	$C_{23}H_{24}ClNO_3$
Formula weight(g/mol)	397.88	397.88
Temperature(K)	110	293(2)
Wavelength(Å)	1.54178	0.71073
Crystal system	monoclinic	Orthorhombic
Space group	C2	$P2_12_12_1$
a(Å)	17.783(5)	9.179(7)
b(Å)	9.6993(19)	14.046(11)
c (Å)	12.796(3)	16.222(12)
α(°)	90	90
β(°)	107.868(10)	90
γ(°)	90	90
V(Å^3)	2100.6(8)	2092(3)
D_{calc}(g/cm^3)	1.258	1.264

Z	4	4
Crystal size(mm)	0.564×0.126×0.119	0.600 x 0.400 x 0.180
Reflection collected	34940	10112
Goodness-of-fit on F^2	1.037	0.829
Final *R* indices(I>2σ(I))	R_1=0.0359, wR_2=0.0879	R_1 = 0.0379, wR_2 = 0.0693
R indices(all data)	R_1=0.0427,wR_2=0.0915	R_1 = 0.0718, wR_2 = 0.0742

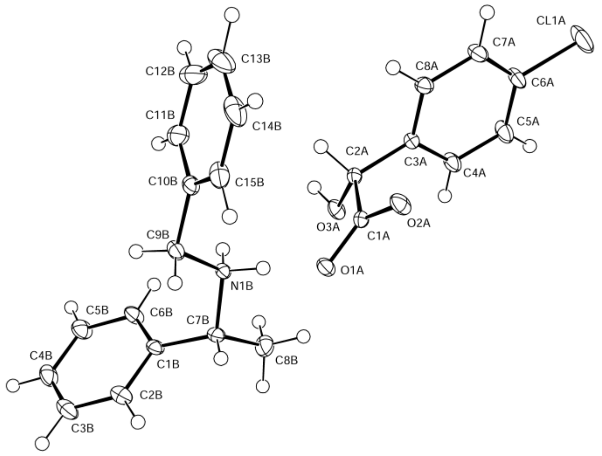

(a)

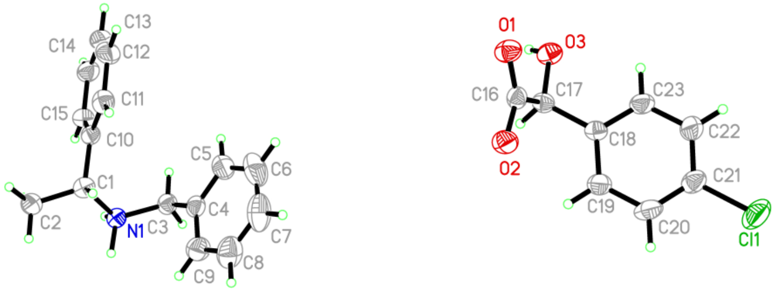

(b)

Figure S1. Atomic-numbering Schemes of (*R*)-(-)-4-ClMA·*(R)*-(+)-BPA (a) and (*S*)-(+)-4-ClMA·*(R)*-(+)-BPA (b)

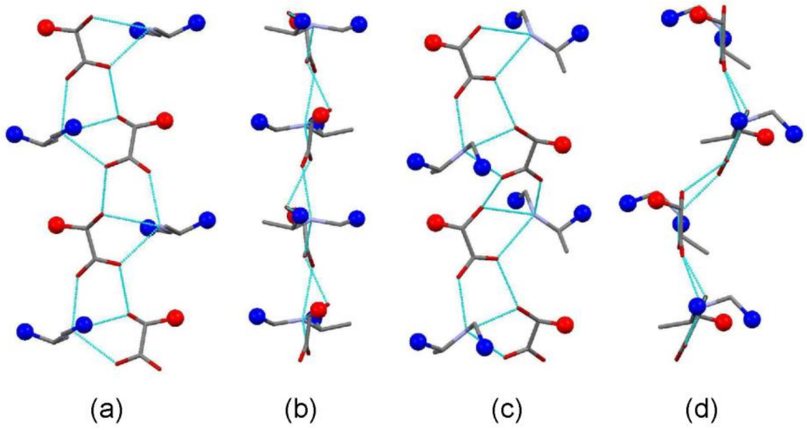

Figure S2: The H-bonding network in the less soluble salt (a and b) and more soluble salt (c and d).The red parts represent carboxylate anions of 4-ClMA and the blue parts represent ammonium cations of BPA

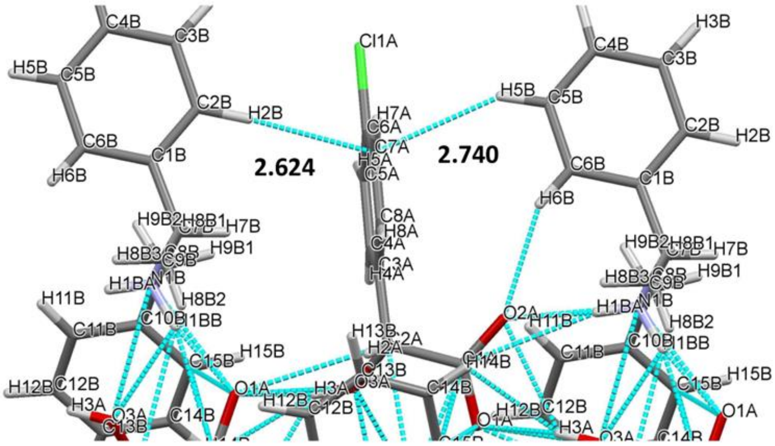

Figure S3. The CH/π interactions within hydrogen column of less soluble salt.

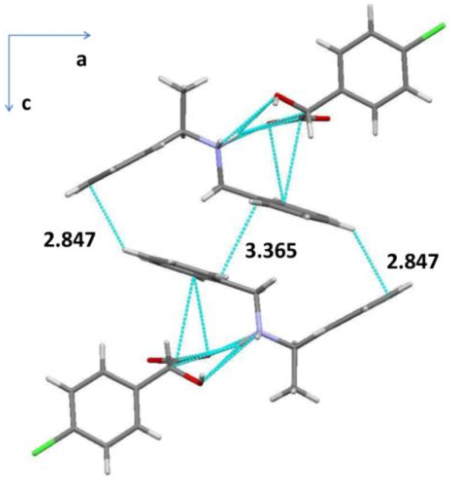

Figure S4: The CH/π interactions between adjacent hydrophobic layers of less soluble salt.Viewed from b-axis

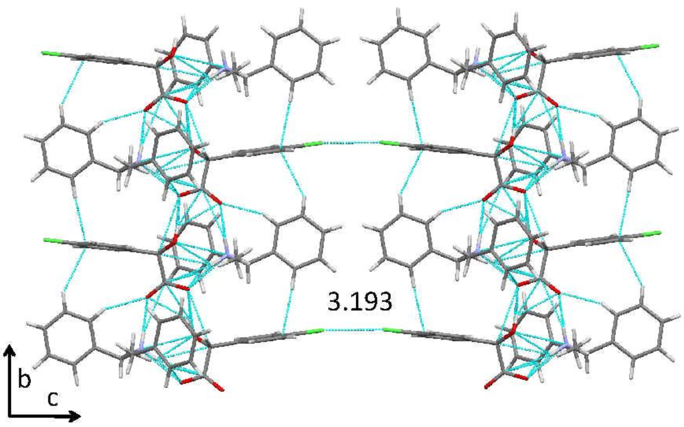

(a)

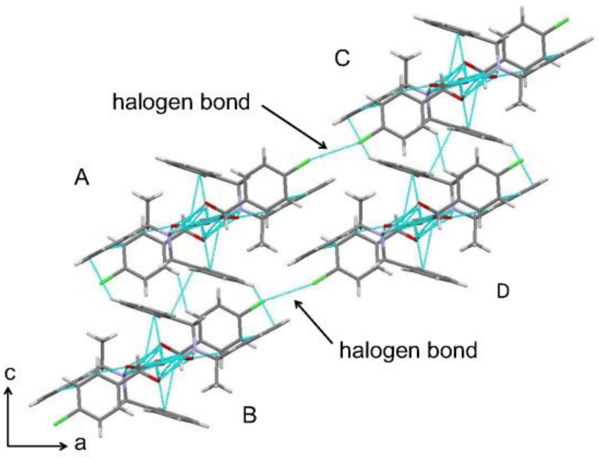

(b)

Figure S5. The Cl…Cl halogen bond between adjacent hydrogen bonding net work columns and the view of adjacent four columns from b-axis in the less soluble salt (a)viewed from a-axis; (b)viewed from b-axis

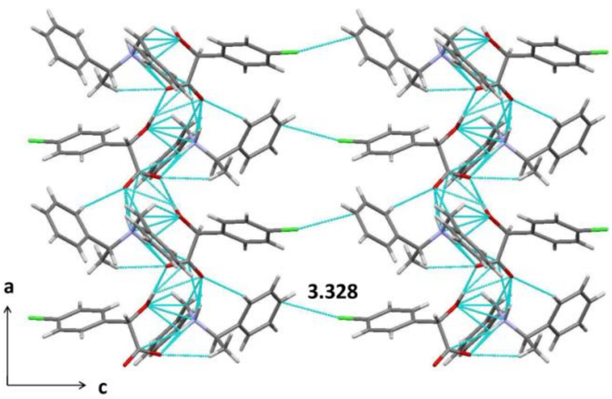

(a)

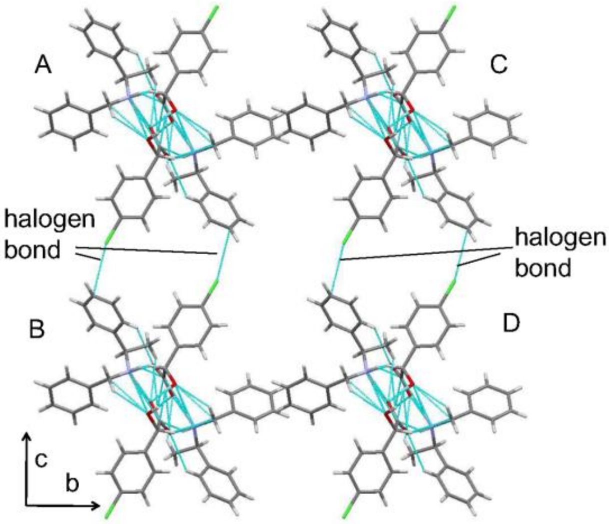

(b)

Figure S6 The Cl/π halogen bonds between columns and the view of adjacent four columns from a-axis in the more soluble salt(a)viewed from b-axis; (b) viewed from a-axis

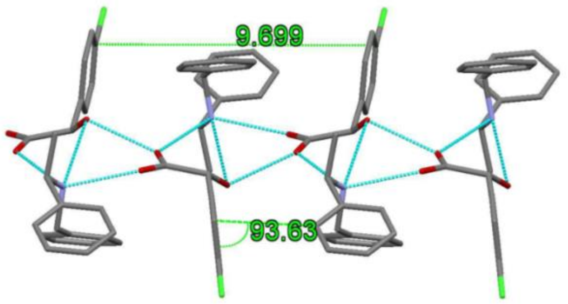

(a)

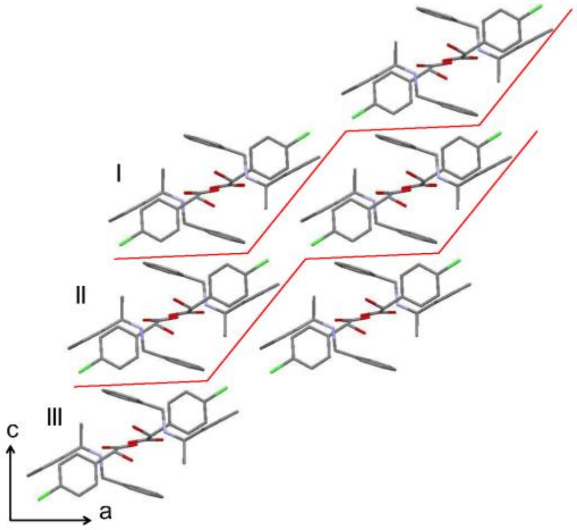

(b)

Figure S7. Packing mode of the less soluble salt in hydrophobic region;(a) The distance of benzene rings; (b) the packing mode of hydrophobic layers （viewed from b-axis）

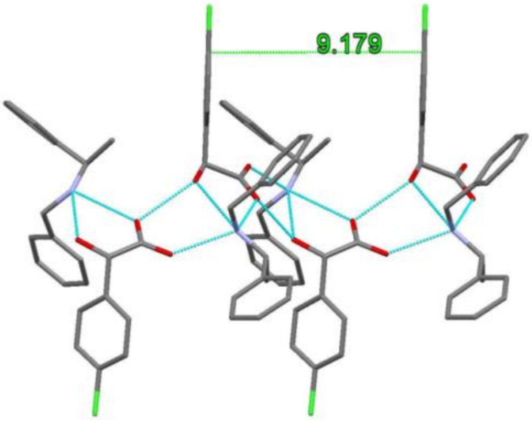

(a)

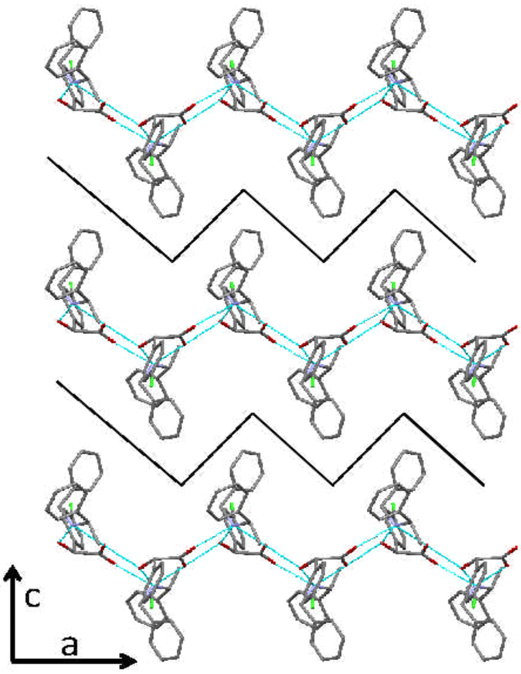

(b)

Figure S8. packing mode of the more soluble salt in hydrophobic region; a, The distance between benzene rings; b, the packing mode of hydrophobic layers（viewed from b-axis）
